# Metabolomic Analysis of Diet-Induced Type 2 Diabetes Using UPLC/MS Integrated with Pattern Recognition Approach

**DOI:** 10.1371/journal.pone.0093384

**Published:** 2014-03-26

**Authors:** Hui Sun, Shuxiang Zhang, Aihua Zhang, Guangli Yan, Xiuhong Wu, Ying Han, Xijun Wang

**Affiliations:** Department of Pharmaceutical Analysis, Key Lab of Metabolomics and Chinmedomics, National TCM Key Laboratory of Serum Pharmacochemistry, Heilongjiang University of Chinese Medicine, Harbin, China; Instituto de Investigación Sanitaria INCLIVA, Spain

## Abstract

Metabolomics represents an emerging discipline concerned with comprehensive assessment of small molecule endogenous metabolites in biological systems and provides a powerful approach insight into the mechanisms of diseases. Type 2 diabetes (T2D), called the burden of the 21^st^ century, is growing with an epidemic rate. However, its precise molecular mechanism has not been comprehensively explored. In this study, we applied urinary metabolomics based on the UPLC/MS integrated with pattern recognition approaches to discover differentiating metabolites, to characterize and explore metabolic pathway disruption in an experimental model for high-fat-diet induced T2D. Six differentiating urinary metabolites were found in the negative mode, and two (2-(4-hydroxy-3-methoxy-phenyl) acetaldehyde sulfate, 2-phenylethanol glucuronide) of which were identified involving the key metabolic pathways linked to pentose and glucuronate interconversions, starch, sucrose metabolism and tyrosine metabolism. Our study provides new insight into pathophysiologic mechanisms and may enhance the understanding of T2D pathogenesis.

## Introduction

The prevalence of diet-induced obesity is increasing globally, and posing significant health problems for millions of people in tne world. Diet-induced obesity is a major contributor to the global pandemic of type 2 diabetes (T2D). Typical civilization disease, particularly T2D, represents one of the most significant global health problems because it is associated with a large economic burden on the health systems of many countries [1]. The WHO predicted an estimated future number of 366 million affected individuals in 2030 [2]. The burden of T2D is growing worldwide and with it a more desperate need for better tools to detect, diagnose and monitor the disease is required. Metabolomicss, defined as ‘the quantitative measurement of the dynamic multiparametric metabolic response of living systems to pathophysiological stimuli or genetic modification’, is increasingly being applied to the study of disease, especially in metabolic disease [3]. New platform metabolomics, focused on a holistic investigation of living systems to external stimuli based on the global metabolite profiles in biological samples, provides variation of whole metabolic networks for characterizing pathological states, as well giving mechanistic insight into the biochemical effects of the drugs [4]. Metabolomics technologies bring a wealth of opportunity to develop new biomarkers which are important tools for identifying diseases, predicting their progression and determining the effectiveness, and doses of therapeutic interventions [5].

Metabolomics may be assumed that in individuals with T2D many metabolic pathways are likely to be affected and presumably play a role in their overall metabolic dysfunction. Thus, the identification of new biomarkers and pathways can improve the characterization of pathophysiological alterations associated with T2D [6]. Understanding the biochemical networks will help to clarify diabetes etiology, and should foster the discovery of new biomarkers of disease risk and severity. We have previously reported an analysis of targeted quantitative metabolomics, where we have shown that many known and novel observations of metabolic changes may be discovered using such a metabolomics approach [7]–[10]. The method has the power to identify perturbations of the body's metabolic homeostasis and thereby offers access to markers of metabolic pathways that are impacted by the disease [11]–[13]. By utilizing this comprehensive biochemical profiling approach, we seek to identify metabolites with different concentrations in T2D, and thereby allowing new insights into the pathophysiological progression of this important metabolic disease.

Various analytical techniques, with multivariate data analysis, such as partial least squares-discriminant analysis (PLS-DA) have been applied in metabolomics-based metabolism studies [14]. UPLC coupled with MS has become one of the widely applied techniques in metabolomics owing to its high sensitivity and reproducibility [15]–[20]. Herein, urinary metabolomics based on UPLC-MS was applied to investigate the metabolic profiles and potential biomarkers in a rat model of T2D, which may facilitate understanding the pathological changes of T2D and obtain a systematic view of dissection of mechanisms of T2D.

## Materials and Methods

### Chemicals and reagents

Acetonitrile (HPLC grade) was purchased from Dikma Technology Inc. (Dima Company, USA). Deionized water was purified by theMilli-Q system (Millipore, Bedford, MA, USA). Formic acid (HPLC grade, FA) was purchased from honeywell Company (USA). Leucine enkephalin was purchased from Sigma-Aldrich (MO, USA). High fat emulsion was prepared by our laboratory. Briefly, lard 20 g, methyl thiouracil 1 g, cholesterol 5 g, sodium glutamate 1 g, sugar 5 g, fructose 5 g, propylene glycol 30 ml were mixed and added water volume to 100 ml and prepared for high fat emulsion (see Ref 21).

### Ethics Statement

Our study was carried out in strict accordance with the recommendations in the Guide for the Care and Use of Laboratory Animals of the Heilongjiang University of Chinese Medicine. The protocol was approved by the Committee on the Ethics of Animal Experiments of the Heilongjiang University of Chinese Medicine (Permit Number: CEAE-HUCM-0126104). All efforts were made to minimize suffering.

### Animal handling

Male Wistar rats (weighting 180–220 g) were supplied by GLP Center of Heilongjiang University of Chinese Medicine (Harbin, China). The room temperature was regulated at 25±1°C with 40±5% humidity. A 12-h light/dark cycle was set, free access to standard diet and water. The animals were allowed to acclimatize for 7 days prior to dosing and putted in the metabolism cages during the urine collection periods specified below. After acclimatization, animals were randomly divided into the control and model groups. Wistar rats were treated with a high fat emulsion (10 ml/kg) by ig for 10 consecutive days to induce T2D (see Ref 21, 22). The rats in the control group were treated with 0.9% saline in the whole procedure for 10 consecutive days. On the last day, rats were deeply anesthetized and then sacrificed. Blood was collected from the abdominal aorta, plasma and serum were separated via centrifuged at 6000 rpm for 20 min at 4°C. The serum was used for biochemical assay according to the manufacturer's instructions of commercial kits. The activities and levels of fasting serum glucose (FSG), triglycerides (TG), total cholesterol (TC), malondialdehyde (MDA), superoxide dismutase (SOD), free fatty acid (FFA), and rate constant for plasma glucose disappearance (KITT) were determined by using commercially available kits. All procedures completely complied with the manufacture. All animal care and experimental procedures were performed in compliance with the Ethical Committee of Heilongjiang University of Chinese Medicine. All efforts were made to ameliorate suffering of animals.

### Sample collection and preparation

All rats of each group were housed in metabolic cages (1 per cage). Urine was collected daily (at 6:00 a.m.) from metabolism cages at ambient temperature throughout the whole procedure and centrifuged at 13,000 rpm at 4°C for 5 min, and the supernatants were stored frozen at −80°C until analysis. All these samples were thawed at room temperature before analysis and centrifuged at 13,000 rpm for 5 min. An aliquot of 5 uL was injected for UPLC/MS analysis after filtered through a 0.22 um membrane filter.

### Metabolic profiling

#### Chromatography

UPLC/ESI-Q-TOF/MS was used for the global analysis of urine samples. Chromatographic analysis was performed in a Waters ACQUITY UHPLC system controlled with Masslynx (V4.1, Waters Corporation, Milford, USA). An aliquot of 6 μL of sample solution was injected onto an ACQUITY UPLC BEH C_18_ column (50 mm×2.1 mm, 1.7 μm, Waters Corporation, Milford, USA) at 35°C, the flow rate was 0.5 mL/min, and injection volume was 2 μL. The optimal mobile phase consisted of a linear gradient system of (A) 0.1% formic acid in water and (B) 0.1% formic acid in acetonitrile, 0–1 min, 99–90% A; 1–4 min, 90–80%A; 4–6 min, 80–65%A; 6–9 min, 65-1%A; 9–11 min, 1%A; 11–11.5 min, 1–99%A, 11.5–13 min, 99% A. In addition, the QC sample was used to optimize the condition of UPLC-Q-TOF/MS, as it contained most information of whole urine samples. A QC sample was operated every 6 urine samples to evaluate stability during sequence analysis. Whenever one sample injection was finished, a needle wash cycle was done to remove the remnants and prepare for the next sample. In addition, the eluent was transferred to the mass spectrometer directly, that is, without a split.

### Mass spectrometry

The mass spectrometry was operated by electrospray ionization in the negative ionization mode. The eluent was introduced into the high-definition mass spectrometer (Waters Corp., Milford, USA) analysis, and the optimal conditions of analysis were as follow: the source temperature was set at 110°C, desolvation gas temperature was 350°C, cone gas flow was 50 h, desolvation gas flow was 600 L/h; the capillary voltage was 2.3 kV, the sampling cone voltage was 35 V, microchannel plate voltage was 2450 V and extraction cone voltage was 3.0 V. The data acquisition rate was set to 0.14 s/scan, with a 0.1 s inter scan delay. Data were colected in centroid mode from 100 to 1000 Da. For accurate mass acquisition, a lock-mass of leucine enkephalin at a concentration of 0.2 ng/mL was used via a lock spray interface at a flow rate of 100 μl·min-1 monitoring for negative ion mode ([M+H]^−^ = 556.2615) to ensure accuracy during the MS analysis.

### Multivariate data analysis and data processing

UPLC-MS raw data were imported into the MassLynx ™ software (Waters Corp.) for peak detection and alignment. Intensity of each ion was normalized with respect to the total ion count to generate a data matrix that consisted of the retention time, m/z value, and the normalized peak area. Multivariate data matrix was analyzed by EZinfo software 2.0 (Waters Corp., Milford, USA). All the variables were pareto-scaled prior to PLS-DA. Here, PLS-DA were used to process the acquired UPLC-MS data. In the PLS-DA modeling, endogenous metabolites that contribute to the classification were identified in loading plots, which showed the importance of each variable to the classification. Student's t-test was performed to identify features with differential abundances across groups.

### Identification of metabolic pathway

Biomarkers of interest were extracted from PLS-DA loading plots based on their contribution to the variation and correlation within the data set. With regard to the identification of potential biomarkers, the ion spectrum was matched with the structure message of metabolites acquired from biochemical databases, such as HMDB, http://www.hmdb.ca/; KEGG, http://www.genome.jp/kegg/; METLIN, http://metlin.scripps.edu/; Chemical Entities of Biological Interest (http://www.ebi.ac.uk/Databases/); MassBank, http://www.massbank.jp/; Scripps Center for Mass Spectrometry (http://masspec.scripps.edu/index.php) and Lipidmaps (http://www.lipidmaps.org/). The reconstruction and pathway analysis of potential biomarkers was performed with MetPA software based above database source.

## Results

### Multivariate statistical analysis of rat urine

**Figure 1:**
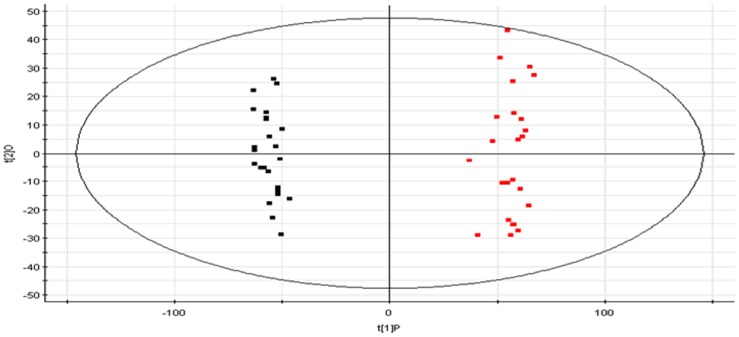
PLS-DA plot derived from the UPLC/MS profiles of rat urine samples demonstrating separation of control group (black) and T2D group (red) rats.

**Figure 2:**
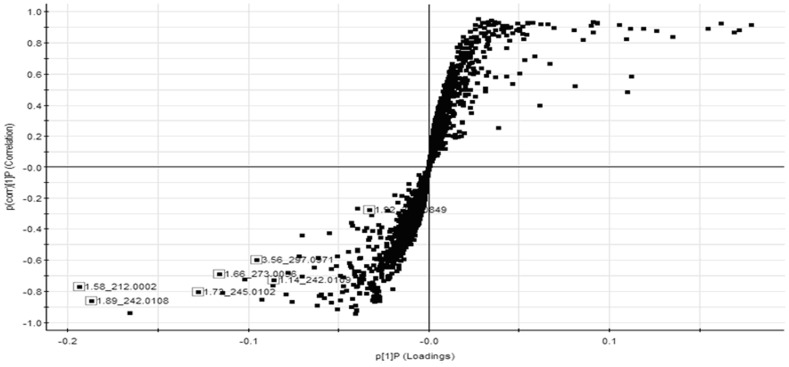
S-plot of PLS in rat urine samples represents the impact of the metabolites on the clustering results. Urine samples from control group and T2D group rats were subjected to UPLC/MS. The PLS model was then used to generate a loadings S-plot showing ions important to the clustering of samples. Box data points indicate that ions most responsible for the variance in the score plot.

The biochemistry parameters of the control group and T2D group were summarized in the Table 1. The biochemical results observed in the T2D group did show significant difference compared to the control group. In this study, a PLS-DA method was established and employed to identify biomarkers which were related to T2D development. As Fig. 1, there is a distinguished classification between the clustering of the control and T2D groups. According to the results of S-plot, a total of 6912 variable ions (Fig. 2) were significantly different between the control and model groups. Finally, 6 of them in negative mode were identified by searching MS and MS/MS fragments in metabolites database, and finally confirmed by commercial standards (table S1).

**Table 1:** Biochemistry results of rat by diet-induced type 2 diabetes.

Group	FSG (mmol/l)	TG(mmol/l)	T-CHO(mmol/l)	MDA(nmol/ml)	SOD(U/ml)	FFA(μmol/l)	KITT	Urine (ml/12 h)
Control	4.36±0.61	0.33±0.09	1.55±0.45	11.56 ±5.77	84.83±23.58	987.35±216.04	0.69±0.30	12.93±4.08
Model	23.63±6.77***	0.69±0.49*	2.87±1.05**	17.33 ±6.33*	50.16±15.68**	1299.65±348.90*	0.37±0.25*	48.88±11.68***

Note: FSG, fasting serum glucose; TG, triglycerides; TC, total cholesterol; MDA, malondialdehyde; SOD, superoxide dismutase; FFA, free fatty acid; KITT, rate constant for plasma glucose disappearance

* significant difference from control at p<0.05;

** Significant difference from control at p<0.01;

*** Significant difference from control at p<0.001.

### Identification of metabolite candidates

The information including the retention time, the exact mass and the ms/ms data were supplied by the robust UPLC-MS platform. The precise molecular mass was determined within a reasonable degree of measurement error using Q-TOF, and the potential element composition, degree of unsaturation and fractional isotope abundance of the compounds were also obtained. Metabolite identification was conducted with high resolution MS and MS/MS fragments, as well as database analyses. We searched for the presumed molecular formula in the ChemSpider, Human Metabolome Database, KEGG, and Small Molecule Pathway Database to confirm possible chemical compositions. According the protocol described above, six endogenous metabolites were identified and summarized in table S1.

### Biomarker network and metabolic pathway reconstruction

**Figure 3:**
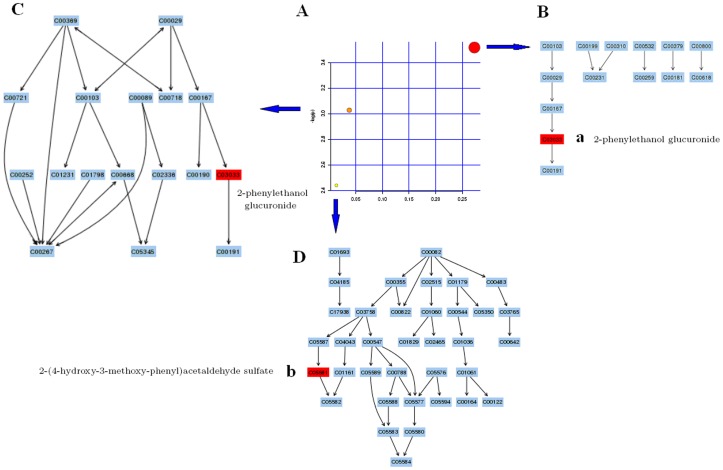
A systemic view of metabolic pathways that associate with T2D in this study, providing a disease specific picture of human physiology. Identifying network pathway by MetPA software (A). Putative metabolic pathways of pentose and glucuronate interconversions (B), starch and sucrose metabolism (C), and pyrimidine metabolism (D) were inferred from rat urine of intermediates during substance metabolism. The map was generated using the reference map by KEGG. Red denotes affected metabolites related to the pathway. a, 2-phenylethanol glucuronide; b, 2-(4-hydroxy-3-methoxy-phenyl)acetaldehyde sulfate.

The related pathways of the biomarkers were investigated by searching the KEGG and HMDB, and a network of some biomarkers was established. Metabolic pathway analysis with MetPA revealed that potential biomarkers are mainly involved in the pathway of pentose and glucuronate interconversions, starch and sucrose metabolism, and tyrosine metabolism that changed specifically in the setting of T2D (Fig.3). Two distinct metabolites (2-phenylethanol glucuronide, 2-(4-hydroxy-3-methoxy-phenyl)acetaldehyde sulfate) identified from these pathways were involved in T2D development, which indicated that dysfunction of multi-pathways were involved in the pathological process of T2D. The detailed construction of the metabolism pathways with higher score was shown in Fig.3. Results suggested that these target pathways showed the marked perturbations over the time-course of T2D and could contribute to development of T2D.

## Discussion

Metabolomics is a rapidly evolving discipline that involves the systematic study of endogenous small molecules that characterize the metabolic pathways of biological systems [23]. It has been studied extensively in human diseases, and has resulted in significant advances in the understanding of the pathophysiology of diseases. Diabetes represents one of the most important global health problems and approximately 90% of patients with diabetes have T2D, and its incidence remains highest in the world [24]. Fortunately, metabolomics has introduced new insights into the pathology of diabetes as well as methods to predict disease onset and has revealed new biomarkers. Recently, a variety of biomarkers reflecting T2D pathologies have been developed to gain new insights into metabolic pathways and pathophysiological mechanisms [25]. Because some of the processes identified above may result in metabolic “signatures” in the urine which would be useful for T2D diagnosis as well as therapeutic responsiveness. Although great effort has been put forth to uncover the complex molecular mechanisms exploited in the pathogenesis of T2D disease, satisfactory explanation remains to be discovered.

In our study, an UPLC–MS-based urine metabolomics approach coupled with multivariate statistical methods provide a powerful approach to clearly differentiate patients with T2D from matched controls and identify the potential biomarkers. Urine samples collected were analyzed by UPLCESI-QTOFMS operating in negative ionization mode. The mass to charge ratio (m/z) and retention time and abundance data generated were subjected to PLS multivariate data analysis. The loading S-plot generated from PLS is a convenient way of visualizing those ions with the highest contribution to the separation between control and T2D in relation to their correlation to the model. PLS-DA is a well-established supervised multivariate statistical analysis method which has been widely used in metabolomic studies. Results indicate that PLS-DA revealed a very good visual separation between the T2D and control samples. Interestingly, 6 distinct metabolites identified from these pathways, many are in various stages of progress at the T2D. For simplicity, only parent compounds are shown in Table S1. Further study of these metabolites may facilitate the development of non-invasive biomarkers and more efficient therapeutic strategies for T2D. Further investigations are also underway to clarify the precise pathogenesis why T2D induced these results. Furthermore, it is noteworthy that 2 metabolites together are important for the host response to T2D through metabolism pathways. Using functional analysis and the KEGG pathway database, we identify several biologically relevant metabolic pathways which are altered in this disease. T2D related metabolites were tightly correlated with pentose and glucuronate interconversions, starch and sucrose metabolism, pyrimidine metabolism, and tyrosine metabolism network that are strongly associated with T2D development. These biochemical changes are helpful to understand the key features of T2D. In addition, these metabolic features provided useful clues for future mechanism exploration and identification of therapeutic targets of T2D.

Emerging metabolomicss provides a powerful platform for discovering novel biomarkers and biochemical pathways to distinguish between diseased and non-diseased status information and improve diagnostic, prognostication and therapy [26]. As emerging platforms in the biomedical arena, metabolomics can make use of multivariate statistical analysis to search for disease-related potential biomarkers and metabolic pathways [27], [28]. Metabolomic network has led to the integration of metabolites associated with the caused perturbation of multiple pathways, most specifically in elucidating mechanisms of disease progression and for biomarker discover. The results not only indicated that urine metabolomic methods had sufficient sensitivity and specificity to distinguish T2D from healthy controls, but also have the potential to be developed into a clinically useful diagnostic tool, and could also contribute to a further understanding of disease mechanisms. Additionally, this unbiased technique, particularly and uniquely associated with the disease, has greatly enhanced our ability to identify novel pathways that are potentially involved in T2D pathogenesis. It is hoped that further integration of this techniques will yield a more comprehensive understanding of T2D disease etiology and the biological pathways.

## Conclusions

Emerging high-throughput metabolomics technologies have been widely applied, aiming at the discovery of candidate biomarkers for disease and may help to understanding the mechanism of T2D occurrence on the metabolic level. Herein, we illustrate how metabolomics can be utilized to explore the mechanisms of T2D which affect different ‘key pathway’. T2D is one of the most common diseases in the world, but currently it is difficult to determine the precise pathophysiology. Here, we applied the metabolomics approach based on the UPLC/MS to systematically investigate T2D. Interestingly, 3 distinct pathways such as pentose and glucuronate interconversions, starch and sucrose metabolism, and tyrosine metabolism *etc*. were found associated with T2D according to ingenuity pathway analysis. Based on our findings, it is suggested that metabolomics approach is highly effective in aiding biomarker identification of T2D. The continuous, dynamic, and noninvasive detection of metabolites in the urine of T2D rats was successfully demonstrated, and will increase our understanding of the pathophysiological processes involved and help us to identify potential biomarkers to develop new therapeutic strategies.

## Supporting Information

Table S1 **A list of potential urinary biomarkers of urine samples from type 2 diabetes.**
(DOC)
