# Supplementary material for: Metabolomic Analysis of Diet-Induced Type 2 Diabetes Using UPLC/MS Integrated with Pattern Recognition Approach
Source: PLoS One. 2014 Mar 26;9(3):e93384. doi: 10.1371/journal.pone.0093384 (PMC3966886; doi:10.1371/journal.pone.0093384)
Supplement: Table S1 — A list of potential urinary biomarkers of urine samples from type 2 diabetes. (DOC) [file pone.0093384.s001.doc]

**Table S1**. A list of potential urinary biomarkers of urine samples from type 2 diabetes.

| **No** | Retention time | Measured mass | Elemental composition | Identified metabolites | P-value |
| --- | --- | --- | --- | --- | --- |
| 1 | 1.89 | 242.0108 | C_9_H_9_NO_5_S | N-acetyl-2-carboxy benzenesulfonamide | 0.00 |
| 2 | 1.58 | 212.0002 | [C_4_H_7_NO_7_P](http://www.chemspider.com/Molecular-Formula/C4H7NO7P) | 4-phospho-L-aspartate | 0.01 |
| 3 | 1.66 | 273.0056 | [C_6_H_8_O_10_P](http://www.chemspider.com/Molecular-Formula/C6H8O10P) | 3-dehydro-L-gulonate 6-phosphate | 0.01 |
| 4 | 1.72 | 245.0102 | C_9_H_10_O_6_S | 2-(4-hydroxy-3-methoxy-phenyl)acetaldehyde sulfate | 0.01 |
| 5 | 1.92 | 338.0849 | C_15_H_17_NO_8_ | 6-hydroxy-5-methoxyindole glucuronide | 0.02 |
| 6 | 3.56 | 297.0971 | C_14_H_18_O_7_ | 2-phenylethanol glucuronide | 0.03 |
